# Criteria for patient selection and indication for intracorneal ring segments in keratoconus

**DOI:** 10.1186/s40662-024-00379-0

**Published:** 2024-03-26

**Authors:** Alfredo Vega, Jorge L. Alió

**Affiliations:** 1 Grupo Miranza, Alicante, Spain; 2 Universidad Miguel Hernandez de Elche, Alicante, Spain; 3 Hospital Virgen de los Lirios de Alcoy, Alicante, Spain

**Keywords:** Keratoconus, Intracorneal ring segments, Keratoconus review, Corneal degenerations

## Abstract

**Background:**

Keratoconus is an ectatic, progressive corneal disorder characterized by alterations in the morphology of the corneal tissue that leads to limitation of visual function of the patient. Intracorneal ring segments (ICRS) are small synthetic devices that are implanted in the corneal stromal in order to regularize the morphology of the tissue therefore improving the visual function and the quality of life of the patients.

**Main text:**

The present narrative review summarizes the main scientific articles developed by the authors in relation to the clinical outcomes and long-term results of ICRS in the treatment of keratoconus. It was found that those patients that benefit the most from this surgical intervention are those that have the most severe form of keratoconus. Additionally, patients with good visual function, those with more than 0.9 in the decimal scale are at risk of losing visual acuity after ICRS implantation. In relation to long-term results, scientific investigations published by the authors demonstrate that ICRS is a stable procedure after long period of time in terms of vision, refraction, and topographic variables in those patients with stable keratoconus. However, in patients with keratoconus and signs of progression, ICRS may not have the capability of halting the progression of the disease. Using artificial intelligence to guide ICRS implantation provide better clinical outcomes and improvement in corneal higher-order aberrations in patients with keratoconus in comparison to those treated using the commercial nomogram of implantation.

**Conclusions:**

ICRS is a safe surgical procedure in the treatment of keratoconus. Patients that benefit most from the surgery are those with a significant visual impairment. ICRS should not be considered in patients with good visual function because of the risk of losing lines of vision. Long-term follow-up demonstrate stability of the clinical outcomes in patients with stable keratoconus although ICRS may not have the ability of halting the progression of the disease. New technologies based artificial intelligence improved the indications and the clinical outcomes of keratoconus patients treated with ICRS.

## Background

Keratoconus is included within a group of diseases known as corneal ectatic disorders. It is characterized by the progressive thinning of the corneal stroma, leading to tissue irregularity and a consequent negative impact on the patient's visual function [1]. From the point of view of its classification, keratoconus can show a wide range of involvement that goes from minimal corneal geometry alterations that are only identified by corneal topography and that do not present any clinical manifestation to several surface and tissue alterations with an important repercussion on the visual function that limits the quality of life of the patient who suffers from it. Today, there are many classifications that are used in clinical practice to determine the severity of keratoconus. However, many of them are obsolete or fundamental and are based on isolated parameters without considering other factors that are closely related to the pathology [2–4]. Therefore, it is important to have a modern, standardized, reproducible classification that is easily accessible to the clinician to offer the best therapeutic option to the patient with keratoconus in addition to the evaluation of the follow-up and the results of the treatments that are applied. In 2011, our research team published for the first time a scientific article in which different clinical and morphological parameters were integrated to classify keratoconus [5]. In this publication, we aim to propose a better approach of different treatments that are available for keratoconus population using different analysis technqiues [6].

In relation to the treatment of keratoconus, there are currently different treatment modalities ranging from contact lens adaptation, corneal collagen cross-linking (CXL), intracorneal ring segment (ICRS) implantation and different modalities of corneal transplantation as penetrating or lamellar keratoplasty [6]. From these, ICRS can be defined as small elements of synthetic material that are implanted deep into the corneal stroma in order to change the morphology of the tissue [7]. The idea of using intracorneal elements to change the cornea morphology is not new and already in the 1960s Blavatskaia et al. were able to demonstrate in experimental studies that the implantation of discs and rings of corneal tissue in the stroma of rabbit corneas were capable to modify the refractive power of the eye [8]. These investigations were followed by studies of Zhivostovsky et al. and Vishenevetsky et al. who, together with the results obtained by Blavatskaia et al., demonstrated that the refractive modifications obtained were directly proportional to the thickness of the implant and inversely proportional to its diameter [8, 9]. The main limitation found by these researchers was that the type of material and the design of the rings and segments caused extrusion shortly after being implanted in the cornea. The concept of intrastromal corneal ring was first proposed in 1978 by Reynolds et al. [10]. The first implants consisted of full 360° rings whose primary purpose was the correction of myopic refractive errors. During the 1980s, various controlled experimental and preclinical studies were carried out to improve the biocompatibility of materials and implant design. In 1991, the first studies were carried out in non-functional eyes of humans, where the effectiveness of these devices in the correction of refractive errors was demonstrated [9]. However, the success of this surgical technique for the correction of refractive errors was soon eclipsed by the rapid advancement, popularity, and good results obtained with excimer laser corneal refractive surgery. Nevertheless, ICRS implant technology found a new horizon as an alternative in the treatment of corneal ectatic disorders. In the year 2000, Colin et al. reported for the first time the results of ICRS implantation in the treatment of patients with keratoconus [11]. Since then, several authors have studied and demonstrated the ability of this surgical technique in improving the corneal geometry and quality of life in patients with corneal ectatic disorders.

In terms of complications, implanting ICRS in keratoconic patients is considered to be a safe surgical procedure mainly due to the advent of the femtosecond technology that provides more precise and predictable size and depth of the stromal tunnels. Among the surgical related complications, they are usually related to an inadequate depth of the stromal channels, segment decentration or asymmetric position of the segment within the tunnels [12]. The most severe surgical related complication is corneal perforation which usually occurs during the rotational movement with the manual dissector. Complications related to femtosecond laser assisted technique usually are mild, like suction ring lost, subconjunctival hemorrhage and just in less than 0.6% of the cases a corneal perforation may be observed [13]. In terms of postoperative complications, the most feared complication after this surgical technique is infectious keratitis; although, it has been reported to be less than 0.1% of the cases when dissecting the tunnels using the femtosecond laser assisted technique [14]. Extrusion and migration of the segment may also be seen after ICRS implantation although this is more often observed when using the mechanical technique [12, 14]. The percentage of ICRS explantation reported in the literature is quite variable and ranges between 1% and 30% [15]. In the study carried out by Coskunseven et al. [13], the authors found an ICRS explantation rate of 5.7%. Similarly, Ferrara et al. [16] assessed more than a thousand cases implanted with ICRS and found that complications requiring ICRS explantation were present in around 4% of the cases. In a recent study conducted by Nguyen et al., the authors found an explantation rate of 6.1% [17]. On the other hand, Piñero and coworkers reported that the percentage of ICRS explantation after mechanical dissectors was 18%, and 13% when using the femtosecond laser assisted technique [18].

Even though rare, in some cases, severe photic phenomena, recurrent epithelial defect, stromal inflammation, corneal melting and infectious keratitis may appear and in these cases segment explantation should be performed. During the postoperative period, white deposits within the stromal tunnel can often be seen. Even when its incidence has been reported to be as high as 60%, these channel deposits do not induce any optical or structural alteration and are considered to be completely benign [19].

The objective of this narrative review is to propose the criteria for the selection of patients with keratoconus who may be susceptible of treatment with ICRS, taking into account the scientific evidence published by the authors as the main criteria.

## Main text

### Implantation of intracorneal ring segments based on the RETICS classification

In 2011, our research group carried out a study in which a classification of keratoconus was proposed taking into account different topographic, aberrometric, and biomechanical parameters, among others, and which were correlated with the patient's visual acuity [5]. Because it was a multicenter study in which different health facilities participated within the framework of health research network defined as “Red Temática de Investigación Cooperativa en Salud (RETICS)”. The classification was called the RETICS classification [3].

Subsequently, in 2013, a retrospective, multicenter, interventional study was carried out where 611 eyes of 357 patients with keratoconus were included, with a mean age of 35.15 ± 11.62 years and who underwent ICRS surgery [6]. ICRS indication was based on keratoconus diagnosis according to Rabinowitz topographical patterns [1]. Keratoconus diagnosis was based on corneal topography and slit-lamp observation. In all cases, preoperative findings characteristic of keratoconus was confirmed; that is, corneal topography revealing an asymmetric bow-tie pattern with or without skewed axes and at least one keratoconus sign on slit-lamp examination, such as localized stromal thinning, conical protrusion of the cornea at the apex, Fleischer ring, Vogt striae, or anterior stromal scarring. The number, thickness and arc length of the ICRS were selected according to the manufacturer nomograms. Corneal incision was placed in the steepest meridian taking into account the corneal topography. The patients were classified into five different groups taking into account the degree of spectacle corrected distance visual acuity (CDVA) in decimal scale as described in the RETICS classification: Grade I, patients with CDVA 0.90 or better; Grade II, patients with CDVA equal to or better than 0.60 and worse than 0.90; Grade III, patients with CDVA equal to or better than 0.40 and worse than 0.60; Grade IV, patients with CDVA equal to or better than 0.20 and worse than 0.40; and Grade Plus, patients with CDVA worse than 0.20. Additionally, success and failure indices were defined with the purpose of determining the efficacy of the surgical technique. Success was defined by those cases that presented the following characteristics 6 months after ICRS implantation:

- Increase of one or more corrected or uncorrected lines of vision.
- Decrease of two or more diopters in the spherical equivalent.
- Reduction of at least one micron in high-order corneal aberrations or coma-like aberrations.

On the other hand, the failure criteria were the following:

- Decreased one or more corrected or uncorrected lines of vision.
- Increase of two or more diopters in the spherical equivalent.
- Increase of at least one micron in high order corneal aberrations or coma-like aberrations.

The data obtained in the preoperative and postoperative visits: 24 h and at months 1, 3 and 6, were taken into account for the analysis of the results.

In relation to the results, we were able to observe that all the patients presented a significant improvement in uncorrected visual acuity at 6 months, regardless of the degree of keratoconus (*P* < 0.05). However, when analyzing the changes observed in CDVA, patients with the mild form of keratoconus, those classified as Grade I, presented a significant loss (*P* < 0.01) of CDVA 6 months after ICRS implantation. In all other patients, a significant improvement (*P* < 0.05) in the vision was observed after the surgical procedure (Table 1).

**Table 1:** Changes in corrected distance visual acuity (CDVA) from preoperative to postoperative 6 months (M) after intracorneal ring segment implantation

CDVA	Preoperative	Postoperative 6 M	*P* value
Grade I	0.05 ± 0.05 (0.05 to − 0.10)	0.08 ± 0.20 (0.40 to − 0.11)	< 0.01
Grade II	0.15 ± 0.10 (0.22 to 0.08)	0.14 ± 0.20 (0.50 to − 0.11)	0.04
Grade III	0.35 ± 0.51 (0.40 to 0.26)	0.26 ± 0.20 (1.00 to 0.00)	< 0.01
Grade IV	0.57 ± 0.06 (0.70 to 0.42)	0.30 ± 0.20 (1.30 to 0.00)	< 0.01
Grade Plus	1.10 ± 0.06 (0.01 to 0.15)	0.42 ± 0.25 (1.30 to 0.00)	< 0.01

Additionally, in the present study, the loss of corrected lines of vision after ICRS implantation was analyzed, considering the severity of the disease based on the visual limitation of patients with keratoconus. We were able to observe that patients classified as Grade I had almost a 40% risk of losing two or more lines of corrected vision after the surgical procedure (Table 2). Likewise, it should be noted that the patients classified with the most severe form of the disease, Grades IV and Plus, were those who presented the greatest benefit in relation to the improvement in corrected visual acuity and, in consequence, those who presented fewer loss of lines of vision (Table 2).

**Table 2:** Percentage of patients that lost two or more lines of corrected distance visual acuity (CDVA) 6 months after intracorneal ring segment implantation

Keratoconus	Lost ≥ 2 lines CDVA ( %)
Grade I	37.83
Grade II	20.68
Grade III	9.45
Grade IV	4.65
Grade Plus	3.70

In relation to what was defined as success and failure indices, we noted that the success and failure rates of the ICRS implant are directly related to the visual limitation that patients have at the time of the surgical procedure. Thus, 85% of patients classified as Grade Plus will gain at least one corrected line of vision 6 months after ICRS implantation, while only 13.5% of patients classified as Grade I, will do so. In the same way, more than half of the patients classified as Grade I will lose at least one corrected line of vision after surgery, while only 11.1% of the patients classified as Grade Plus will present this loss (Table 3).

**Table 3:** Percentage of patients that gain or lost one line of corrected distance visual acuity (CDVA) 6 months after intracorneal ring segment implantation

Keratoconus	Success CDVA (%)	Failure CDVA (%)
Grade I	13.5	51.0
Grade II	49.4	29.8
Grade III	54.0	18.9
Grade IV	81.3	9.3
Grade Plus	85.1	11.1

The results of the present study clearly demonstrate that when analyzing the efficacy of the ICRS implantation and considering the degree of visual limitation that patients have before surgery, the patients with the greatest probability of success are those with the worst visual acuity at the time of the surgical procedure. Additionally, there is clear evidence of the poor results achieved in patients whose visual function is not compromised and therefore ICRS surgery should not be considered a proper indication in those keratoconus cases with good vision before surgery (patients with more than 0.9 in decimal scale). Therefore, nowadays we recommend ICRS implantation for keratoconus treatment in those patients with spectacle CDVA worse than 0.9 in decimal scale, poor patient motivation to wear contact lenses, or contact lens intolerance. Additionally, other specific considerations to avoid implantation are the ones recommended in the manufacturer nomograms, e.g., corneal pachymetry safety limit that suggest avoiding implantation of segments of 350 μm of thickness in corneas with less than 580 μm at sites where the tunnel will be dissected. It is also not recommended to implant ICRS in those keratoconic patients with severe central corneal scarring or with evidence of previous corneal hydrops.

### Long-term results of ICRS implantation in keratoconus patients

ICRS implantation for the treatment of keratoconus is a surgical technique that has been used for the last 20 years approximately [11].

We analyzed the results of the ICRS surgery for keratoconus treatment after having followed up the patients for at least 5 years [20, 21].

In the first study, the authors analyzed 51 eyes of 35 patients with stable keratoconus with a mean age of 29.00 ± 8.84 years. Stability was defined as patients with no more than 1.00 D of change in the mean keratometric readings over the last 12 months [20]. The follow-up period was 5 years in all cases. We were able to observe a statistically significant improvement in both uncorrected and corrected visual acuity 6 months after ICRS implantation (*P* < 0.05). The improvement found 6 months after surgery in the visual variables did not undergo significant changes throughout the 5 years of follow-up (*P* > 0.30). There are several articles published in the literature in which the visual results of the ICRS implant in patients with keratoconus have been analyzed and most of them report an improvement in vision after surgery [22–25]. Additionally, some authors who have carried out analysis of the results after long periods of time have found stability of the surgical technique at the end of the follow-up period [22, 26–28].

The same behavior was observed in relation to the refractive parameters. Specifically, we found a statistically significant (*P* = 0.04) reduction in spherical equivalent 6 months after ICRS implantation, with no significant change (*P* = 0.57) observed over the subsequent 5 years. Once again, in the studies carried out and in which the implantation of segments has been evaluated after long periods of time, they have also observed stability of the refractive variables [22, 26, 27].

**Fig. 1:**
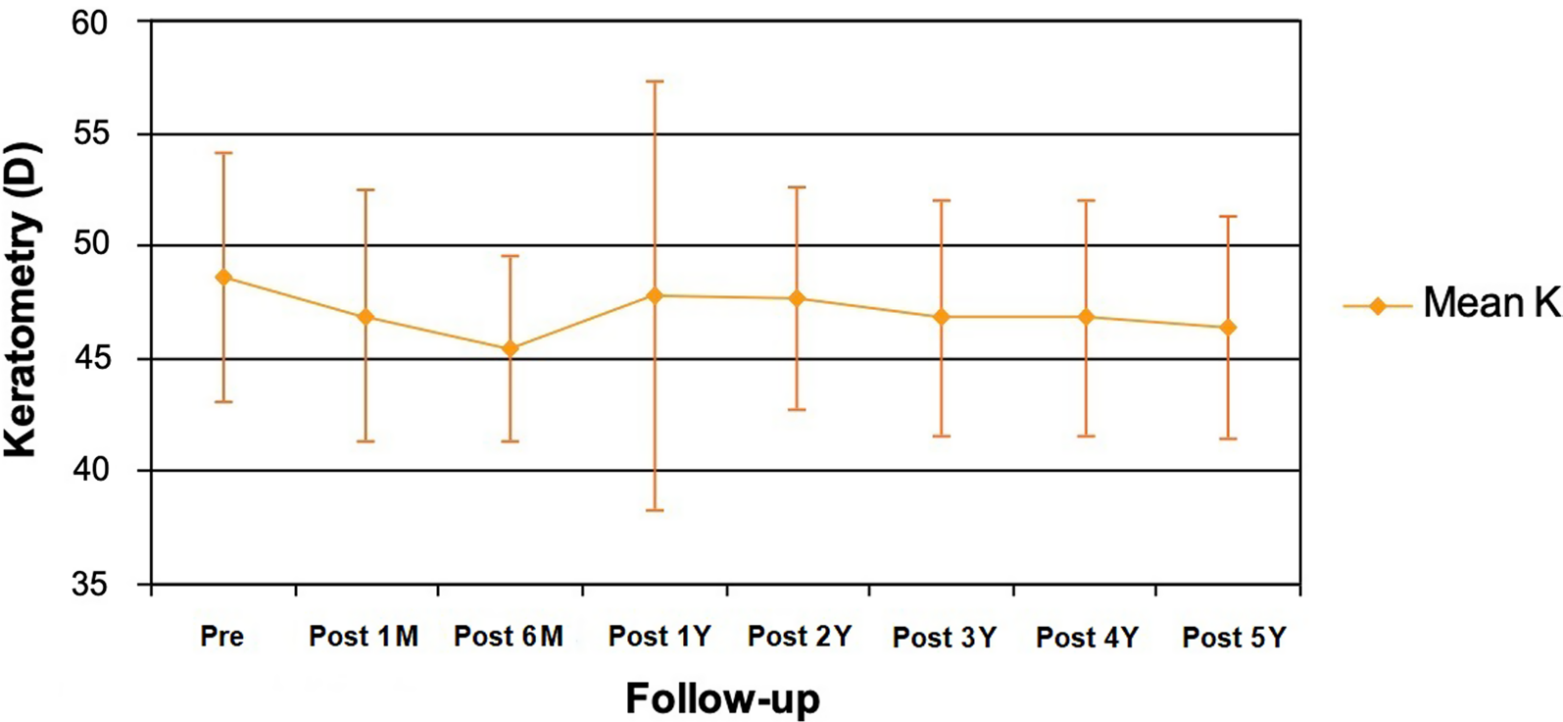
Evolution of mean keratometric reading (mean K) in diopters (D) from the preoperative period (pre) through 5 years of follow-up. M, month; Y, year

In relation to the topographic changes, we found a statistically significant reduction (*P* ≤ 0.02) both in the flattest, steepest and mean keratometry (Kmean), 6 months after surgery, without significant changes throughout the follow-up period. Regarding Kmean, we observed a statistically significant (*P* < 0.01) reduction of 3.24 D 6 months after ICRS implantation. Afterwards, a minimal regression in Kmean, of less than 1.00 D, was observed between 6 months and 5 years after the surgical procedure. It should be noted that the changes observed in Kmean between 6 months and 5 years were not statistically significant (*P* = 0.39; Fig. 1).

In different studies that have analyzed the topographic changes in patients with keratoconus after ICRS implantation, they have reported an improvement in the keratometric variables immediately after surgery and that, in addition, these changes remain without significant changes after long follow-up periods [22, 23, 25–27].

This improvement was observed in the visual, refractive and topographic variables 6 months after ICRS implantation. The consequent stability throughout the 5 years of follow-up demonstrate that the benefits provided by the surgical procedure in keratoconus patients with no sign of progression remain stable and without significant changes after long follow-up periods. Finally, we must remember that the patients included in this study were patients in whom visual, refractive, and topographic stability had been confirmed for at least 12 months prior to ICRS implantation. Therefore, these results indicate that we can expect the changes found immediately after ICRS implantation to remain unchanged after long periods of follow-up in a population with stable keratoconus. However, what we cannot affirm is that the ICRS implantation can stop the progression of the disease, since for this it would be necessary to evaluate the surgical technique in a population with the progressive form of the disease.

Along the same line by analyzing the long-term result after ICR implantation in the treatment of keratoconus, we decided to study patients with keratoconus with signs of progression.

For this purpose, we carried out a study in which a total of 18 cases of patients with progressive keratoconus were included [21]. Patients analyzed in the current scientific investigation were between 19 and 30 years old (mean age of 25.75 ± 3.59 years) and were followed during a period of 5 years after ICRS implantation. Progression of the disease was defined when one or more of the following criteria was documented over a 6-month period:

1. Increase in the steep or Kmean reading ≥ 0.75 D in a period of 6 consecutive months.
2. Increase in the refractive cylinder ≥ 1.00 D in a period of 6 consecutive months.
3. Increase the refractive sphere ≥ 1.00 D in a period of 6 consecutive months.
4. Reduction of two or more corrected lines of vision in a period of 6 consecutive months.

To determine the progression of the cases, two preoperative visits were documented: the first, 6 months before ICRS implantation, and the second, just before the surgical procedure.

**Fig. 2:**
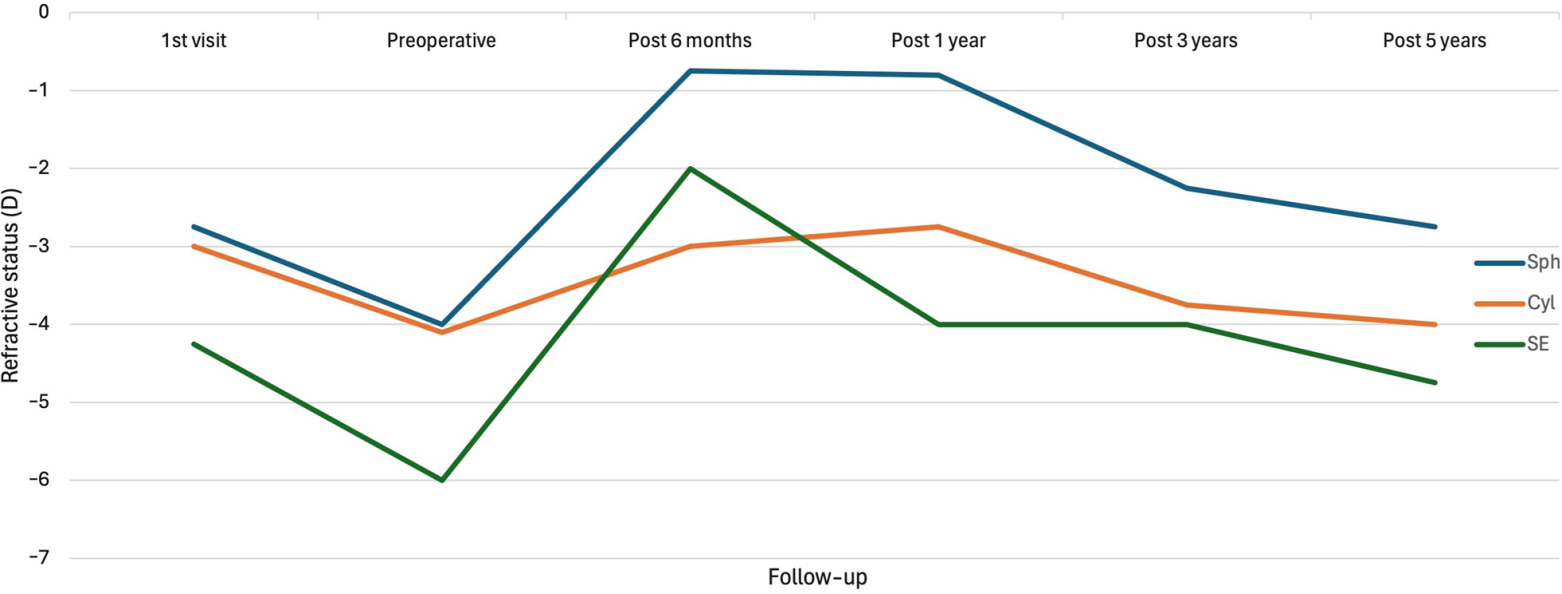
Evolution of the refractive variables, sphere (sph) cylinder (cyl) and spherical equivalent (SE) in diopters (D) during the follow-up period

Here, we were able to observe a reduction in both vision and refractive variables (cylinder and spherical equivalent) during the 6 months prior to ICRS implantation, which confirmed the progressive nature of the cases we were analyzing. Subsequently, 6 months after surgery, an improvement in all the variables, both visual and refractive, was observed, which coincided with most of the series in which an improvement in vision and refraction was reported after ICRS implantation for the treatment of patients with keratoconus [23, 24, 26, 29, 30]. However, there was a regression or worsening of the effect during the follow-up between 6 months and 5 years. During the latest period, the refractive and visual variables returned to levels that are similar to those found during the preoperative assessment (Fig. 2).

**Fig. 3:**
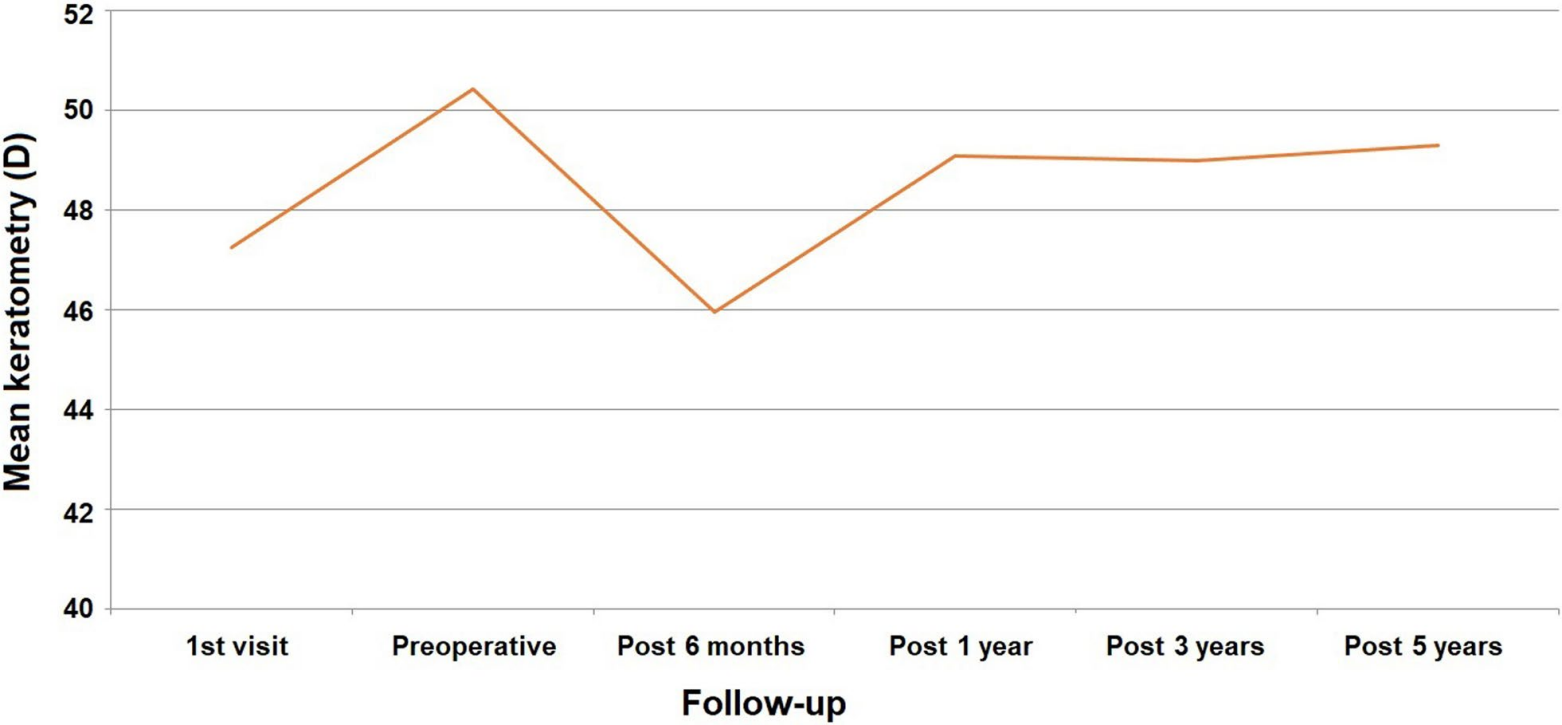
Evolution of mean keratometric reading in diopters (D) during the follow-up period

Regarding the keratometric readings, we were able to observe a significant increase of 3.17 D in the Kmean values when we compared the first and preoperative visits (*P* < 0.01), which confirmed the progressive nature of the cases under analysis. Six months after the ICRS implant, we found a statistically significant reduction of 4.40 D in the Kmean (*P* < 0.01). Despite the significant reduction in the keratometry values at 6 months, there was a significant change in that effect throughout the follow-up period, where a regression of 3.36 D was observed in the Kmean between the visit at 6 months and the last evaluation at 5 years (Fig. 3).

If we analyze the results of the two studies that were carried out in order to analyze ICRS implantation in keratoconus patients after 5 years of follow-up [20, 21], we must consider that the effect of the ICRS will depend on the stability or progression of the cases present at the time of the surgical procedure. On the one hand, we will have those patients in whom the stability of the disease has been documented and in whom the benefit obtained immediately after the procedure is expected to remain unaltered after long periods of follow-up. While in patients in whom there is clinical evidence of disease progression, the beneficial effect achieved after surgery may be lost over time. Nevertheless, it should be noted that the results of this study must be taken with cautious because of the retrospective nature of the study and the limited number of patients included in the cohort. Moreover, we have to consider that keratoconus is a progressive disease mainly during the first three decades of life. Even when progression of the disease was documented in the aforementioned study [21], we have to consider the possibility of natural stability in some of these cases as the mean age from the studied patients was 25.75 years old.

### Limitations after ICRS implantation in patients with keratoconus

ICRS implantation is an effective surgical technique in improving vision and refraction in patients with keratoconus. Similarly, it is a safe and stable procedure after long periods of follow-up. However, it has certain limitations as can be expected in any type of surgery. We note that patients with good visual acuity are not good candidates for this procedure. Our results show that about 40% of patients with visual acuity of 0.9 in decimal scale or better will present loss of corrected lines of vision after surgery [6]. It is for this reason that from this point of view and based on our scientific publications, this surgical technique should not be considered in patients with good visual function due to the high percentage of patients who will lose vision after surgery.

On the other hand, regarding the long-term results, both our publications and that of most authors have shown that the ICRS implantation provides a long-term benefit in patients with keratoconus. Nevertheless, it is necessary to assess if the cases are stable or have signs of progression at the time of surgery. Thus, our scientific publications show that long-term stability can be expected if the cases are stable, that is, they do not show signs of progression at the time of surgery. On the other hand, if there is evidence of disease progression at the time of ICRS implantation, it is most likely that the benefit effect achieved during the first few months after the procedure will be almost completely lost after 5 years. It is for this reason that we must document the stability of keratoconus before considering treatment with ICRS. In the event that we have doubts or keratoconus is clearly progressing, surgery with ICRS would not be advisable because it has not been shown that this technique alone is capable of halting keratoconus progression.

Recently, our research group conducted an investigation to analyze the changes observed in patients with keratoconus that have overcome ICRS implantation in whom an extrusion had occurred [31]. Specifically, we wanted to analyze changes accounted in keratoconic patients that could be taken as prognostic factors of late extrusion in cases implanted with ICRS. For that aim, we conducted a multicenter study including 23 keratoconic corneas that were implanted with ICRS and these were followed during a period of at least 2 or more years. In all cases, a natural extrusion of one of the segments occurs at least 2 years after the primary procedure. In that study, the average time interval was 5 years. Topographic findings came close to baseline; the Kmean readings before the explantation surgery 48.97 ± 3.47 D and 47.60 ± 3.67 D after explantation (*P* = 0.374). Furthermore, a significant worsening in the refractive cylinder that was just after the implantation ‒2.54 ± 3.40 D changed to ‒3.96 ± 1.72 D just before extrusion (*P* < 0.05). The main factor obtained before ICRS implantation was the severity of the keratoconus grade, keratometric readings, and the visual acuities. After ICRS implantation, the most relevant components were the refractive cylinder, CDVA, and uncorrected distance visual acuity. Corneal aberrations were the main factors in the pre-explantation analysis. From the results of the aforementioned study, we concluded that ICRS can be safely extracted, with a regression of the corneal topographic variables towards to the preoperative level. One of the most important observations from this investigation is that a significant change in the refractive cylinder before the extrusion of the segments might be present before the explantation, suggesting that this parameter can be used as a prognostic factor in such cases [31].

**Fig. 4:**
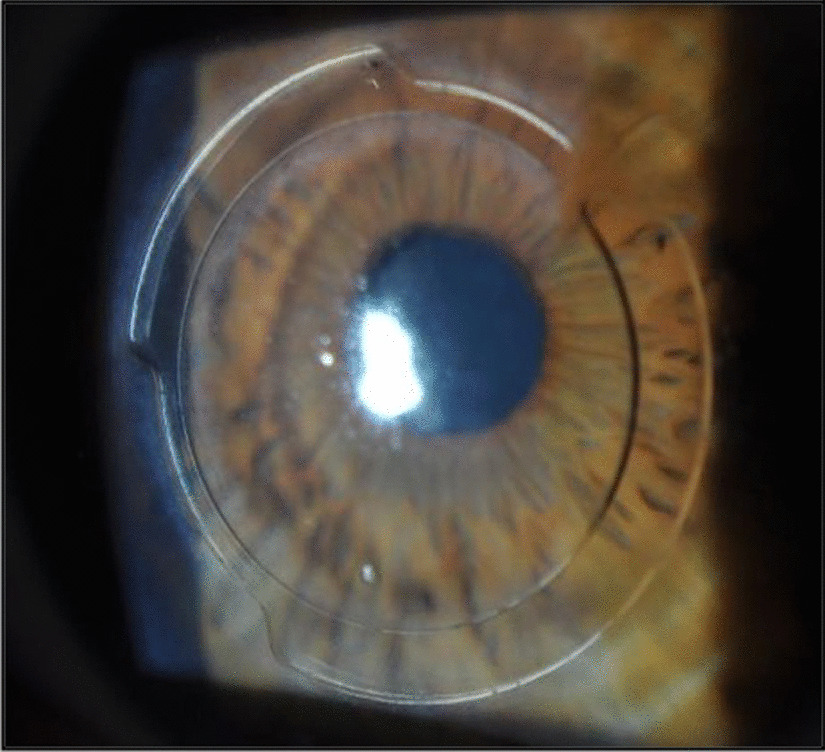
Slit-lamp photograph showing the eye of a patient implanted with the VISUMRING

Along the same lines and regarding limitations of ICRS, the authors published in year 2019 a pilot study evaluating a new design of an asymmetric long arc length intracorneal ring for the treatment of keratoconus [32]. The ring was named as VISUMRING (VR) and was characterized of having an arc length of 353 degrees and two asymmetric sections that can be customized in base width, length, and thickness (Fig. 4).

In this study, we analyzed the visual acuity, refraction and corneal higher-order aberrations of 30 eyes from 26 patients implanted with the VR that were followed with a mean period of 14.7 months. It was found that a significant improvement of both uncorrected and corrected vision was achieved after implantation of the VR. We also observed a significant reduction of more than 7.00 D in the spherical equivalent that correlates well with a significant flattening of the cornea which usually is observed after implantation of long arc length ICRS. Nevertheless, despite of the good clinical outcomes after implantation of the VR in keratoconic patients we found that 5 of the 30 cases needed to have a surgery to explant the VR during the follow-up due to severe focal corneal melting in the area of the incision. We hypothesize that performing a single incision for the purpose of implantation is a clear advantage, the long arc length of VR makes the ends of it fall in the stroma just beneath the incision site, leadings to wound healing alterations that can induce corneal melting and consequently, ring extrusion. This 17% explantation rate observed in our study could be considered a high complication rate and therefore implantation of ICRS with such designs should be avoided until new devices are developed and studies demonstrate their safety.

### Future perspective

We recently carried out a scientific study in which it was possible to demonstrate that the use of artificial intelligence (AI) enhanced the indications of ICRS implantation. Specifically, an artificial neural network (ANN) was created to be used as a guide in ICRS surgery. In the aforementioned study, we aimed to analyze the clinical results of ANN that has been designed for the purpose of improving the predictability of ICRS implantation in the treatment of keratoconus. For that purpose, we compared 40 patients implanted with ICRS. In one of the groups, group A, we included 20 patients with keratoconus that were implanted with the Keraring ICRS (Mediphacos, Belo Horizonte, Brazil); in this group, the selection of the number (1 or 2), arc length, and thickness of ICRS was performed following the manufacturer’s nomograms. In the second group, group B, we included 20 keratoconic eyes that were also implanted with the Keraring ICRS but in this case the selection of the number (1 or 2), arc length, and thickness of ICRS was performed following the recommendation by the artificial neural network. This artificial neural network includes an algorithm that simulates which combinations of segments could provide the best topographic outcome and best corneal optical quality, and thus the best quality of vision, as a function of the Strehl ratio, for the patient [33]. In that study, we found that the spherical equivalent and the keratometric values decreased significantly in both groups. The corrected vision improved from 0.20 ± 0.21 logMAR preoperatively to 0.15 ± 0.20 logMAR postoperatively in the group B (*P* < 0.005), and from 0.26 ± 0.21 logMAR preoperatively to 0.22 ± 0.20 logMAR postoperatively in the group A (*P* < 0.01), with statistically significant differences between the two groups (*P* < 0.05), being better in the group where ICRS were selected using the ANN. We noted that those patients in which ANN was used in order to guide the procedure had better optical quality with a reduction in high-order corneal aberrations after the surgery when compared with those patients guided by the commercial nomogram [33]. AI based on neural networks is a dynamic process in which the system is fed (input) with new information, which improves the response it provides. It is for this reason that increasing the input of tools such as ANN can lead to an improvement in the predictability of the results of surgical techniques such as ICRS implantation and therefore improve the treatment of patients with keratoconus. Nowadays, AI is present in several of our daily activities, and is a technology that will impact every aspect of our society and change the paradigm of health care to our patients.

## Conclusions

ICRS implantation for the treatment of patients with keratoconus is an effective and stable technique. It is necessary to consider that the scientific evidence collected by our research team shows that patients with good visual function are not good candidates for this surgical technique and that the stability of the disease must be documented before considering this surgery. When it comes about ICRS extrusion, it was found that a significant change in the refractive cylinder before the extrusion of the segments might be present before the explantation, suggesting that this parameter can be used as a prognostic factor in such cases. Further improvement in the design of long arc length ICRS should be performed before considering these types of ICRS in order to avoid the high rate of extrusion that accompany those designs. Finally, the use of artificial intelligence will enhance the predictability of the outcomes and therefore the quality of life of patients who overcome ICRS implantation for keratoconus treatment.

## Data Availability

Not applicable.
